# The 15-year trend in adherence to dietary recommendations and ultra-processed food consumption in Italy

**DOI:** 10.3389/fnut.2025.1623827

**Published:** 2025-08-05

**Authors:** Jacopo Niccolò Di Veroli, Sara Capruzzi, Umberto Scognamiglio, Laura Rossi

**Affiliations:** ^1^Council for Agricultural Research and Economics - Research Centre for Food and Nutrition (CREA – Food and Nutrition), Rome, Italy; ^2^Department of Food Safety, Nutrition, and Veterinary Public Health, National Institute of Health, Rome, Italy

**Keywords:** diet quality index, ultra-processed foods, temporal changes, nutrient intake, Italy

## Abstract

**Introduction:**

Diet quality indices are essential tools for evaluating dietary patterns in relation to health and sustainability. This study aims to assess the quality of the Italian diet using the Adherence to Italian Dietary Guidelines Indicator (AIDGI) and World Index for Sustainability and Health (WISH2.0) and to estimate ultra-processed food (UPF) consumption as an indicator of poor diet quality.

**Methods:**

Italian food consumption data from 2005 to 2006 and 2018 to 2020 were sourced from the European Food Consumption Comprehensive Database. The dataset includes a wide range of foods categorized by age and sex; for this analysis, two population groups were considered: adults (18–64 years) and the elderly (65–74 years).

**Results:**

The sample sizes included 2,313 adults and 290 elderly individuals in 2005–2006 and 726 adults and 156 elderly individuals in 2018–2020. AIDGI and WISH2.0 showed scores close to 50% of the theoretical maximum achievable indicating that there is substantial room for improvement in the Italian dietary quality. Older adults, particularly women, exhibited healthier eating habits compared to younger adults and men.

**Discussions:**

A temporal trend was observed, having different characteristics in adults and the elderly. Both AIDGI and WISH2.0 scores indicated a more favorable change over time in the elderly compared to adults (AIDGI: +5.6% in the elderly, −5.9% in adults; WISH2.0: +2.8% in the elderly, −5.1% in adults). Although ultra-processed foods (UPFs) accounted for only 6% of total food consumption by weight in 2018–2020, they contributed to 23% of total energy intake. Despite only a modest increase in their consumption by weight, their percentage of energy intake has nearly doubled compared to 2005–2006. In contrast, the consumption of processed foods (PFs) decreased in terms of weight, from 16 to 11%, while their contribution to energy intake remained relatively stable (~28%).

**Conclusions:**

This study reveals a gradual decline in the quality of Italian diets over time, primarily reflected in the increasing consumption of UPFs and distinct demographic patterns. While overall adherence to dietary recommendations appears relatively stable, this may partly reflect the fact that current guidelines do not explicitly differentiate between UPFs and minimally processed foods—an aspect with important implications for public health.

## 1 Introduction

Diet quality indices are essential tools for evaluating dietary patterns in relation to both health and sustainability. These indices evaluate how closely a diet aligns with established dietary guidelines or specific nutritional frameworks, offering a quantitative measure of diet quality (1). Among the most widely used are the Healthy Eating Index (2), the Mediterranean Diet Score (3), and more recent indices such as the EAT-Lancet Index (4) and the World Index for Sustainability and Health (WISH) (5), which integrate both health and environmental sustainability criteria using the Planetary Health Diet as reference (1). Comparative analyses of these indices provide valuable insights into regional, demographic, and temporal variations in dietary habits, helping to identify areas where targeted nutritional interventions are needed (6). Diet quality indices assess adherence to nutritional recommendations—an important determinant of public health—given their influence on the risk of non-communicable diseases and overall well being. Research consistently shows that adherence to these guidelines remains suboptimal across populations. Various factors influence adherence, including socioeconomic status, cultural dietary habits, nutrition knowledge, and food availability (7). Gender differences have also been noted, with women generally showing higher adherence to dietary recommendations compared to men (8).

From a geographical perspective, Mediterranean countries tend to have higher compliance with plant-based dietary recommendations, consistent with their traditional dietary patterns. In contrast, Northern and Western European countries show a greater consumption of processed foods (PFs) and animal products (9, 10). However, even in Southern Europe, adherence to the Mediterranean diet (MD) has progressively declined—a phenomenon described as dietary westernization (11). Italy is no exception: several studies have documented a significant decline in adherence to the Mediterranean diet (12, 13), although the findings vary depending on the index used. Notably, a study involving 2,869 adults found that only 13% of participants reported high adherence to the Mediterranean diet (14). When comparing Italy with other Mediterranean countries, it is important to highlight that, over the past decade, European, Middle Eastern, and North African nations within the Mediterranean region have generally shown only moderate adherence to the Mediterranean diet (MD) (15). Italy's dietary evolution within the regional context can be better understood through the findings of (11) who analyzed global adherence to the MD between 1960 and 2011. Among the 17 countries classified as Mediterranean, the average Mediterranean Adequacy Index score declined significantly over time, dropping from 3.46 to 2.00. Despite this overall trend, Italy and Portugal were among the few countries that maintained scores relatively close to their initial values, although they too experienced a decline. In 2021, the Adherence to Italian Dietary Guidelines Indicator (AIDGI) was developed to measure how closely the Italian population follows national dietary guidelines (16). When applied to national food consumption frequency data, the AIDGI revealed that 51% of the population did not meet the recommended guidelines (17). The analysis showed excessive intake of processed meats, snacks, sugary drinks, and alcoholic beverages (including wine and beer), alongside insufficient consumption of milk and yogurt, fruits and vegetables, and legumes.

As previously mentioned, Mediterranean countries are increasingly shifting away from traditional dietary patterns, reflecting a broader westernization of food consumption behaviors. A key feature of this shift is the rising consumption of ultra-processed foods (UPFs) (18), which are widely recognized as indicators of poor dietary quality (19).

At European level, Mertens et al. (20) estimated UPF consumption by applying the NOVA classification (18) to food consumption data from the European Food Safety Authority's (EFSA) Comprehensive Food Consumption Database. Their findings showed that the energy contribution from UPFs varied widely across 22 European countries—from 14% in Italy and Romania to 44%in the United Kingdom and Sweden. Additional data on UPF consumption in Italy come from a study involving 8,569 adults and 509 children/adolescents, based on telephone and self-reported interviews (21). This study reported that UPFs contributed 17% of total energy intake in adults and a higher 26% in children and adolescents. Moreover, UPF consumption was associated with several psychosocial factors and eating behaviors, underlining its complex and multifactorial nature.

Against this background, the present study aimed to evaluate the quality of Italian dietary patterns by applying two indices of adherence to dietary recommendations, each offering a complementary perspective: the AIDGI, which reflects national recommendations, and the WISH, which assesses alignment with the Planetary Health Diet, that integrate nutritional and environmental dimensions. This dual assessment provided a broader understanding of the strengths and weaknesses of current eating habits in Italy and allowed for a comparison of Italian dietary patterns with two different gold standards, one national and one international. In addition, the study estimated the consumption of UPFs, analyzing their contribution to total energy and nutrient intake in Italy as indicators of diet quality. The relationship between UPF and adherence to dietary guidelines deserves closer examination as the level of UPF consumption may significantly influence overall diet quality (22). Since AIDGI and WISH2.0 differ in their underlying principles—AIDGI focusing on national dietary recommendations (16) and WISH2.0 integrating both health and environmental sustainability (5)—the way they capture or reflect UPF consumption may vary substantially. Understanding how UPF intake aligns or diverges across these two frameworks can offer valuable insight into the strengths and limitations of each index in capturing critical aspects of modern dietary patterns. Temporal trends were assessed by comparing food consumption data from two national surveys (2005–2006 and 2018–2020), with analyses stratified by gender and age groups (adults vs. elderly). The age groups considered (18–64 and 65–74 years) were opportunistically selected based on their representation in the EFSA Database. These groups were appropriate for the study's objectives, as they allowed for the evaluation of dietary differences between adults and the elderly, while excluding individuals in advanced age, whose dietary patterns may be more strongly influenced by health-related factors (23). A further methodological objective was to adapt the AIDGI for use with food consumption data expressed in grams per day as it was originally developed for categorical frequency data.

The study was guided by the following hypotheses: (i) a progressive decline in the quality of Italian diets over the past 15 years, reflecting the gradual erosion of the Mediterranean diet observed in Southern Europe; (ii) a traditional low but increasing consumption of UPFs in Italy; and (iii) shifts in the consumption of key food groups—such as fruits and vegetables—potentially influenced by national education and promotion campaigns. Finally, this study addressed three specific research questions: (i) what major changes in adherence to dietary recommendations have occurred in Italy over the past 15 years? (ii) Which food groups should be encouraged or reduced to promote health and sustainability? (iii) How applicable is the NOVA classification to Mediterranean countries such as Italy, and to what extent should the concept of UPFs be integratedinto dietary guidelines?

## 2 Materials and methods

### 2.1 Data source

The dataset utilized in this study was sourced from the European Food Consumption Comprehensive Database, which has been developed and maintained by the European Food Safety Authority (EFSA) since 2011 (24). This database provides representative data on the general population, detailing the quantities and varieties of foods consumed across European countries. Specifically, summary statistics on Italian food consumption were obtained for the years 2005–2006 (INRAN SCAI 2005–2006) and 2018–2020 (IV SCAI ADULT 2018–2020) (25). The dataset includes an extensive range of food items, categorized by population subgroups based on age and stratified by sex. For this analysis, two population groups were considered: adults (18–64 years) and elderly individuals (65–74 years). In the 2005–2006 survey, the sample included 2,313 adults (1,068 males and 1,245 females) and 290 elderly (133 males and 157 females), while in the 2018–2020 survey, the sample comprised 726 adults (346 males and 380 females) and 156 elderly (65 males and 91 females). Detailed descriptions of the studies' design, sample, and survey protocol were reported elsewhere (26–28). In brief, the 2005–2006 survey was a cross-sectional study in which households were randomly selected based on a geographical stratification of the national territory, without the application of weighting factors. Food consumption was assessed over three consecutive days using individual estimated dietary records (26). The 2018–2020 survey was designed to align as closely as possible with EFSA's EU Menu methodology, which aims to harmonize food consumption data across EU Member States (28). A nationally representative sample, covering the four main geographical areas of Italy, was obtained through appropriate sampling procedures and with the application of weighting factors. Dietary intake was assessed using two non-consecutive 24-h dietary recalls, conducted at least 2 weeks apart; the 24-h dietary recall was coupled with a Food Propensity Questionnaire to get information on the frequency of consumption of specific foods and food supplements (27). Data collection in 2018–2020 was significantly affected by the COVID-19 pandemic, which led to a temporary suspension of fieldwork, a shift to remote methods, and challenges in participant recruitment—particularly for infants and the elderly—ultimately preventing the achievement of the EFSA-recommended sample sizes for these groups (28). Although the datasets included in the EFSA Comprehensive Database differ methodologically, they remain the only available source for such analyses, as both surveys employed comparable sampling designs, ensured national representativeness, used consistent food classification systems, and validated tools.

### 2.2 The database creation

To assess nutrient intake and determine the proportion of nutrients derived from UPFs, the database of Italian food consumption from EFSA was integrated with the food composition database of the Council for Agricultural Research and Economics (CREA), which provides information on energy, macronutrients, and micronutrients (29). As CREA food composition database was a high-quality and comprehensive source, only a few food items had missing values. In these limited cases, alternative data sources were consulted and cross-checked. If inconsistencies or implausible values were identified, priority was given to values from official databases. This entire procedure was conducted by experts in the field to ensure consistency and accuracy. Specifically, additional data were retrieved from the European Institute of Oncology (IEO) database, the Food Composition Database for Epidemiological Studies in Italy (BDA) (30), and the FoodData Central database of the United States Department of Agriculture (USDA) (31). Subsequently, additional data were sourced from the online database Open Food Facts—Italy (32). Each food item was matched with the composition data that most accurately aligned with its classification in the FoodEx2 Exposure Hierarchy. Missing values were imputed using data from similar food items within the same FoodEx2 category. Furthermore, all food items were categorized according to the AIDGI, WISH, and NOVA classification systems, as detailed in the following sections.

### 2.3 The adherence to Italian dietary guidelines indicator: AIDGI

In this study, adherence to the Italian Dietary Guidelines was measured using the AIDGI, which was specifically adapted for the purposes of the study. The procedure for constructing the indicator was outlined by Scalvedi et al. (16) and was based on the recommended frequency of food consumption, expressed through an ordinal categorical variable (Supplementary Table S1). However, in EFSA's food consumption database, the average daily consumption for each food item and food group was provided. Hence, it was necessary to adapt the construction of the indicator to align with the dataset used in this study. The original qualitative frequency scale (never, less than once a week, a few times per week, once per day, and more than once per day) was converted into estimates of the number of servings consumed. In addition, referring to the Italian Healthy Eating Guidelines (33), which is the gold standard for this adherence assessment, and using the recommended portions expressed in the same guidelines, the standard portion size for each of the 18 food groups included in the AIDGI was calculated as a weighted average based on weekly consumption frequencies recommended by the guidelines for each food. This approach was chosen to ensure consistency with national dietary recommendations and to enable quantitative alignment with the available consumption data. While no formal validation of this adapted method was performed in this study, it preserves the conceptual structure of the original AIDGI and reflects the relative emphasis placed on different food groups in the guidelines.

However, to better align with guidelines for foods that should be consumed daily—such as “fruits,” “vegetables,” “bread, pasta, rice,” and “milk and yogurt”—it was necessary to refine the frequency classification by dividing the “more than once per day” category into two distinct groups: “between once and twice a day” and “more than twice a day.” Thus, multiplying the number of servings by the standard portion size allowed for the assignment of a score for each of the 18 food groups. The scoring system, based on both consumption frequencies and the amount of food consumed in grams per day, is detailed in Supplementary Table S2. Consistent with the original AIDGI version, this study assigned +2 points for consumption aligned with dietary recommendations, 0 points for consumption significantly deviating from recommendations, and +1 points for intake that was close to, but not fully in line with, recommendations. For non-recommended foods, a binary scoring system was applied, with a score of 2 points given only for no consumption, while any intake above zero received 0 points. The overall AIDGI score was then calculated as the sum of the scores across all 18 food groups with a maximum obtainable score of 36.

### 2.4 The World Index for Sustainability and Health (WISH2.0)

To assess how the Italian dietary pattern aligns with global dietary recommendations, the World Index for Sustainability and Health (WISH) was used. WISH is an indicator designed to measure adherence to EAT-Lancet recommendations for a healthy and sustainable diet in the general population (5). This indicator was created to assess the healthiness and sustainability of diets. Thus, seeks to measure two complex multidimensional concepts, diet quality, and environmental sustainability, in a single scoring system. WISH included 13 food groups, with the final score calculated as the sum of scores from each group ranging from 0 (no adherence to the EAT-Lancet recommendations) to 130 (perfect adherence to the EAT-Lancet recommendations). In the context of the EU Horizon PLAN'EAT Project (34) aimed to provide data and recommendations to transform the food system toward healthier and more sustainable dietary behavior, the original WISH index was revised and the WISH2.0 indicator (35) developed. This revision involved the addition of two food groups: alcohol and processed meat, both of which are considered important for public health and sustainability (36, 37). The WISH2.0 index used in this study comprised 15 food groups, with a revised score range from 0 to 150, maintaining the principle that lower scores indicate lower adherence to EAT-Lancet recommendations. All 15 components were categorized based on their healthiness, following the guidelines of the Planetary Health Diet (1), and their environmental impact, as outlined by Clark et al. (38).

Each food group was assigned a score ranging from 0 to 10, where 0 represents non-adherence to the recommended intake, and 10 indicates full adherence. Intermediate values were determined proportionally based on the intake range, assuming a linear relationship between consumption levels and their impact on health and sustainability outcomes.

For protective food groups, this relationship was direct, and the intermediate score was calculated with the following formula:


$$\begin{array}{l} score=10*\frac{observed\;intake-lower\;recommeded\;intake}{recommeded\;intake-lower\;recommeded\;intake} \end{array}$$

On the other hand, for neutral food groups and for those to limit the intake, this relationship was inverse, and the intermediate score is calculated with the following formula:


$$\begin{array}{l} score=\\ 10^{*}\;\frac{\left(upper\;recommended\;intake-reported\;intake\right)-\;\left(observed\;intake-\;recommeded\;intake\right)}{upper\;recommeded\;intake-\;recommeded\;intake} \end{array}$$

The final total WISH2.0 score was calculated as the sum of the scores for all 15 food groups. In addition to the total WISH2.0 score, four sub-scores were calculated corresponding to the adherence to the recommendation of high consumption of healthy foods sub-WISH2.0, low consumption of unhealthy foods sub-WISH2.0, high consumption of low-impacting foods sub-WISH2.0, and low consumption of high impacting foods sub-WISH2.0. The WISH2.0 calculation scoring system and the food items recommended intake were reported in the Supplementary Table S3.

### 2.5 The NOVA classification of food consumed in Italy

Following the procedure described by Mertens et al. (20), the foods consumed in Italy were codified according to the NOVA classification (39). Depending on the level of processing, codes from 1 to 4 have been assigned corresponding to 1 = natural or minimally processed foods; 2 = processed culinary ingredients; 3 = processed foods (PFs); and 4 = ultra-processed foods (UPFs). The nature of the FoodEx2 coding details system did not permit to distinguish if the products were home-prepared or industrially produced; hence, assumptions were made considering products such as fine bakery wares or dairy-based desserts as UPFs (20) For alcoholic beverages, differentiation was carried out including wine and beer in group 3 (PF) and the other alcoholic distilled drinks in group 4 (UPFs) (40).

## 3 Results

### 3.1 The adherence of the Italian food consumption pattern to national dietary guidelines (AIDGI) and Planetary Health Diet (WISH2.0)

AIDGI and WISH2.0 food groups' consumption levels (g or ml/day) are reported in Table 1. Changes were observed in terms of temporal trends considering the 15-year spanning of the two data collections and in terms of population demographic characteristics. In both 2005–2006 and 2018–2020 data collection, the most consumed food groups in terms of grams per day were “fruits” (245 g with large differences by age, 199 g for adults and 292 g for elderly), “vegetables” (226 g), and “bread, pasta and rice” (187 g, with a difference by sex, 163 g for females and 211 g for males), followed by “milk and yogurt” (119 g,133 g for females and 105 g for males) and “beer and wine” (117 g, with a huge difference by sex, 59 g for females and 176 g for males). It was also interesting to note that the consumption of red meat was higher in males (over 70 g) than in females (40–50 g). A similar situation was found in sugary drinks, which was less than half in the elderly compared to adults (29 vs. 67 g).

**Table 1:** Absolute consumption level (g or ml/day) of selected food groups used to calculate AIDIGI and WHIS2.0: temporal changes by sex and by age groups (adults and elderly).

**Year**	**2005–2006**						**2018–2020**					
**Population group**	**Adults**			**Elderly**			**Adults**			**Elderly**		
**Gender**	**Female**	**Male**	**Total**	**Female**	**Male**	**Total**	**Female**	**Male**	**Total**	**Female**	**Male**	**Total**
**Common food groups in AIDGI and WISH2.0**												
Vegetables	210.75	230.52	220.64	225.70	242.41	234.06	205.51	217.34	211.43	232.18	245.60	238.89
Fresh fruits	214.92	198.50	206.71	274.70	260.99	267.85	186.59	194.83	190.71	293.44	338.37	315.91
Milk and yogurt	136.31	110.14	123.23	137.08	109.55	123.32	118.67	103.30	110.99	141.25	98.07	119.66
Dairy products	55.43	67.01	61.22	49.55	56.38	52.97	44.46	57.00	50.73	42.83	51.21	47.02
Poultry	18.56	23.04	20.80	23.47	20.28	21.88	33.75	47.80	40.78	26.45	27.69	27.07
Red meat	55.23	72.70	63.97	51.07	74.17	62.62	41.86	74.30	58.08	39.00	67.29	53.15
Processed and cured met	23.99	36.68	30.34	18.05	26.71	22.38	24.11	33.14	28.63	21.56	24.38	22.97
Fish and fisheries products	44.53	48.39	46.46	38.47	53.53	46.00	44.63	51.93	48.28	34.99	71.42	53.21
Eggs	18.64	24.38	21.51	17.73	24.19	20.96	12.55	17.40	14.98	12.73	18.92	15.83
Legumes	11.34	11.81	11.58	10.79	13.97	12.38	11.10	11.25	11.18	9.26	13.65	11.46
Nuts	0.56	0.69	0.63	0.43	0.42	0.43	6.04	7.62	6.83	3.82	7.84	5.83
Beer and wine	56.73	159.02	107.88	66.33	182.83	124.58	54.32	157.36	105.84	58.19	203.93	131.06
Other alcoholic beverages	1.35	3.88	2.62	0.40	1.42	0.91	3.51	10.35	6.93	0.30	2.60	1.45
**AIDGI—Exclusive food groups**												
Bread, pasta, rice	170.57	223.27	196.92	187.08	232.56	209.82	138.19	194.18	166.19	155.40	194.72	175.06
Potatoes	45.99	53.48	49.74	53.44	58.41	55.93	40.98	52.22	46.60	26.67	41.62	34.15
Sugary drinks	43.02	64.97	54.00	33.12	23.95	28.54	57.44	104.26	80.85	25.69	33.47	29.58
Cakes and sweet snacks	33.69	40.38	37.04	21.61	30.40	26.01	62.10	72.53	67.32	47.94	45.18	46.56
Salty snacks	2.55	1.38	1.97	0.82	0.94	0.88	4.56	4.85	4.71	2.39	5.97	4.18
**WISH2.0—Exclusive food groups**												
Whole grains	38.32	50.46	44.39	37.76	55.97	46.87	32.18	51.02	41.60	33.77	55.17	44.47
Saturated fats	3.07	3.69	3.38	3.23	3.28	3.26	0.93	1.21	1.07	1.06	0.79	0.93
Unsaturated fats	33.97	39.64	36.81	32.91	40.77	36.84	26.55	33.10	29.83	25.77	34.84	30.31
Added sugars	25.67	31.18	28.43	21.71	24.64	23.18	25.02	30.34	27.68	18.26	18.51	18.38

Analysis of temporal trends revealed that some food groups remained relatively stable or showed minor changes. Vegetable intake decreased slightly overall (−1%), particularly among adult males (−6%). Fruit intake increased overall by 5%; however, entirely due to increased consumption among the elderly (+18%), both females (+7%) and males (+30%), while it declined among adults (−8%), especially in females (−13%) compared to males (−2%).

Milk and yogurt consumption decreased by 6% across all age and gender groups, except for elderly females, who showed a slight increase (+3%). Legume consumption declined by 5%, particularly among elderly females (−14%). Whole grain intake declined by 6%, particularly among females (adults −16% and elderly −11%), showed a slight increase (+1%) in adult males. Wine and beer intake rose by 2% overall, however, subgroup analysis revealed decreases in all groups, except elderly males, who exhibited a substantial increase (+12%). Processed meat consumption remained stable (~29 g for adults and 23 g for the elderly), with a slight overall reduction (−1.5%) across all groups, except for elderly females, who showed a substantial increase (+19%).

The food group with the largest increase was nuts, with a 12-fold increase across the population and a nearly 19-fold increase in elderly males (from 0.4 to 7.8 g). Salty snack consumption also increased across all groups. Adult females showed a rise of nearly 80% (from 2.6 to 4.6 g), while elderly males increased their intake by 500% (from 0.9 to 6 g). Overall, salty snack consumption increased by 139% among adults and by 375% among the elderly. Males showed a 393% increase, compared to 135% in females.

Cakes and sweet snack consumption rose by 80% (from 32 to 57 g). Elderly females showed an increase of +122% (from 22 to 48 g), while elderly males increased by +49% (from 30 to 45 g). Sugary drink consumption rose by +27% overall, particularly among adults (+50%) and elderly males (+40%), while elderly females showed a decline (−22%). The consumption of alcoholic beverages other than beer and wine more than doubled over the 15 years. Among adults, both males and females exhibited a similar increase (+165%), while among the elderly, overall consumption increased by 60%, with marked differences between genders (+83% in males and −25% in females).

Among animal-based foods, poultry consumption doubled in adults (+96%, from 21 to 41 g) and increased in the elderly (+24%, from 22 to 27 g). Fish consumption increased by 10% overall, with the highest increase among elderly males (+33%). A decrease was observed only in elderly females (−9%).

Saturated fat consumption showed the largest overall decline (70%, from 3 to 1 g). Unsaturated fat intake decreased by 18% (from 37 to 30 g). Egg intake declined by 27% (from 21 to 15 g), while dairy products decreased by 14% (from 57 to 49 g), with reductions consistent across all subgroups. Red meat intake fell by 12% across most groups, with the exception of adult males, who showed a slight increase (+2%).

All carbohydrate-rich food groups showed a general decline in consumption: bread, pasta, and rice (−16%), whole grains (−6%), and potatoes (−23%). The decrease was particularly pronounced among females, both adults (−19% for bread, pasta, and rice; −16% for whole grains; −11% for potatoes) and the elderly (−17% for bread, pasta, and rice; −11% for whole grains; −50% for potatoes).

### 3.2 The adherence to Italian dietary guidelines indicator: AIDGI

**Figure 1:**
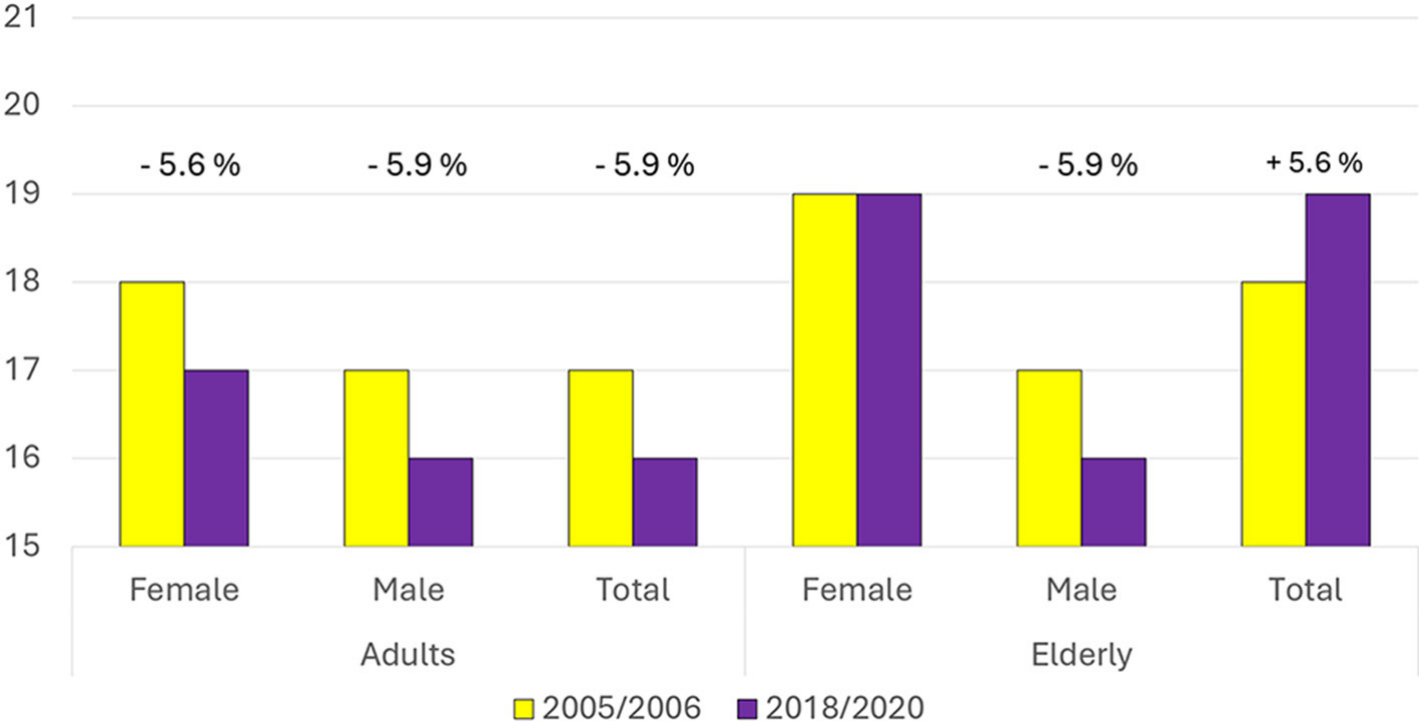
Comparison of the AIDGI index scores between 2005–2006 and 2018–2020 for females, males, and the total population, separately for adults and the elderly. Percentage changes between the two time periods are indicated above each group.

Figure 1 presents the results of the AIDGI calculation, showing that adults had lower adherence to dietary recommendations compared to the elderly, considering the average consumption. In 2005–2006, adults achieved a score of 17 out of 36 (44% of the theoretical maximum), which declined to 16 in 2018–2020—a 5.9% decrease. In contrast, elderly individuals scored 18 out of 36 (50% of the theoretical maximum) in 2005–2006, increasing to 19 in 2018–2020 (+5.6%). Across all time points and age groups, females consistently achieved higher scores than males. For example, males consistently scored 0 points in the red meat category due to exceeding the recommended intake levels.

**Figure 2:**
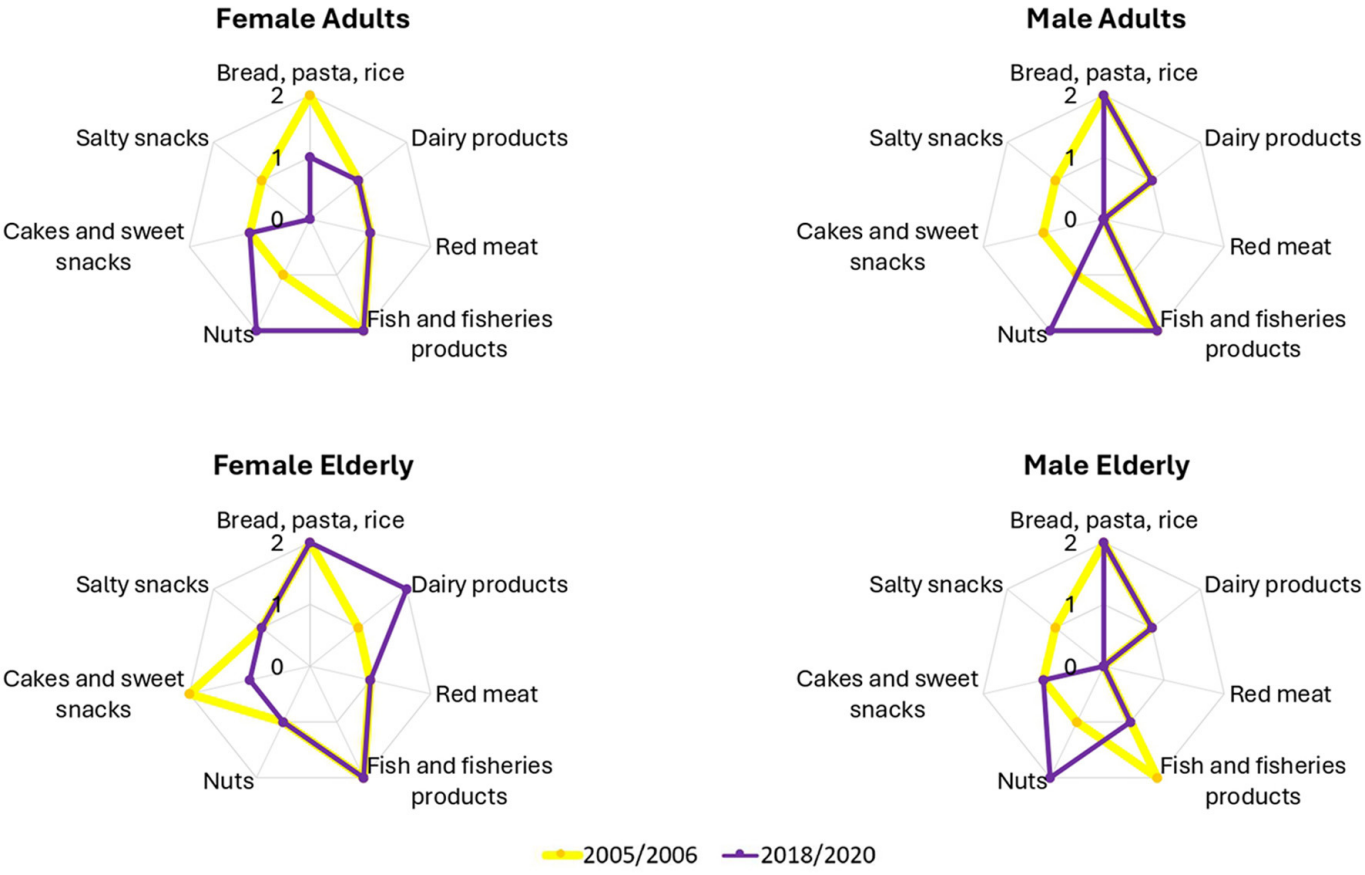
Comparison of AIDGI component scores between 2005–2006 and 2018–2020 for females and males, separate for adults and the elderly. Each radar graph shows the sub-scores for the food items of each demographic group. Only the food items that exhibited changes over time or differences across demographic groups are included.

As shown in Figure 2 and Supplementary Table S4, several food categories—processed meat, beer and wine, other alcoholic beverages, sugary drinks, and legumes—deviated substantially from the recommendations. For these categories, all demographic groups scored 0 out of 2 points in both survey periods. Conversely, potato, poultry, and egg consumption consistently aligned with the recommendations, with all groups scoring 2 points. Bread, pasta and rice, and fish also generally received full scores across population groups.

Among adults, a 1-point reduction in AIDGI score was observed for both sexes over time. Male scores dropped from 17 to 16 (−5.9%) and scores from 18 to 17 (−5.6%; Figure 1). The overall reduction in scores was associated with multiple category-specific changes between the two survey waves. Nut consumption improved, resulting in a 1-point gain for both sexes. However, this was offset by increased intake of salty snacks (1-point reduction for both sexes), higher consumption of cakes and sweet snacks (1-point reduction in males), and reduced intake of bread, pasta, and rice (1-point reduction in females; Figure 2 upper panels).

In the elderly group, males also showed a 1-point decrease in the AIDGI score (−5.9%) between the two time points (Figure 1). This was due to increased nut consumption, which contributed a 1-point gain, being offset by higher intakes of salty snacks and fish and fish products, each resulting in a 1-point loss. Elderly females maintained a stable AIDGI score. A 1-point decrease due to increased intake of cakes and sweet snacks was balanced by a 1-point gain associated with reduced dairy product consumption (Figure 2, lower panels). Nut consumption in this subgroup remained below the recommended level in both periods.

### 3.3 The World Index for Sustainability and Health (WISH2.0)

**Figure 3:**
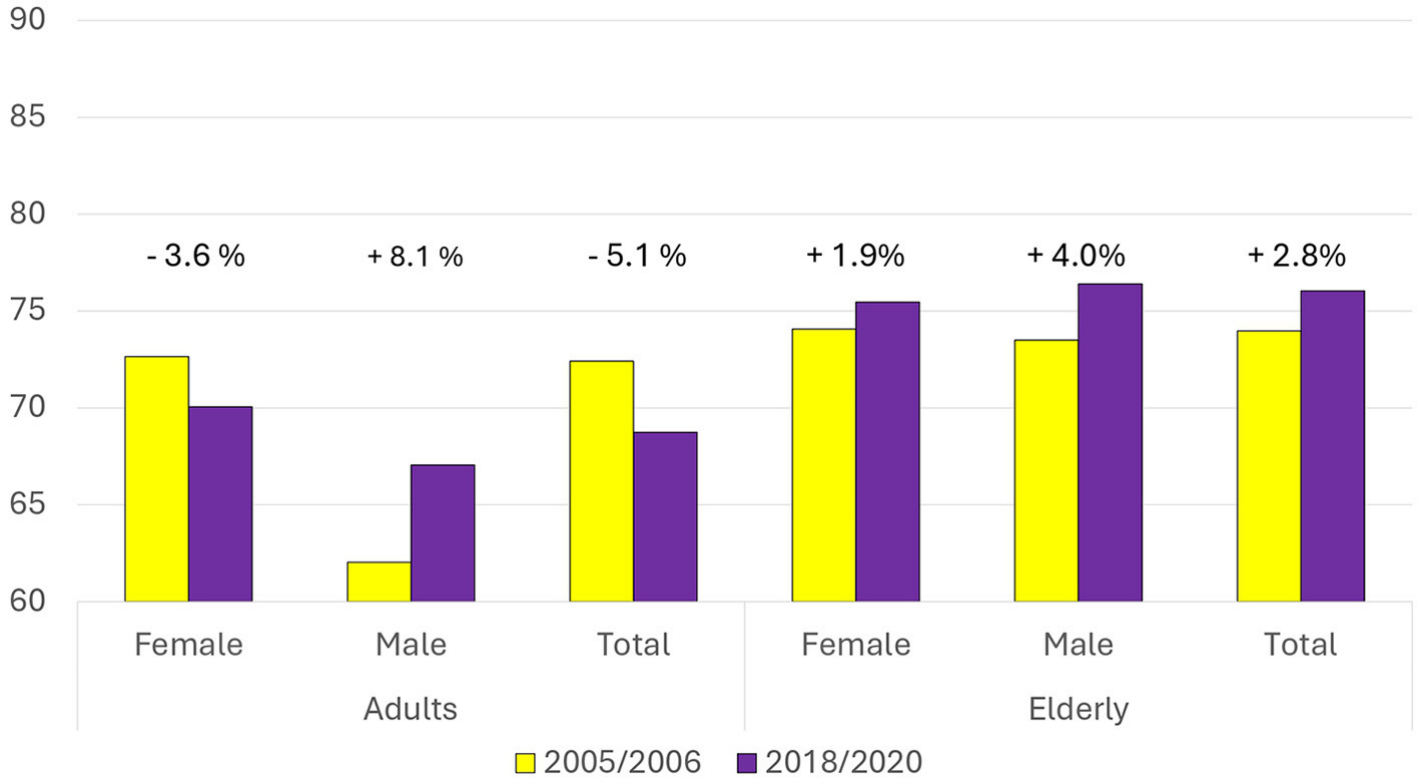
Comparison of the WISH index scores between 2005–2006 and 2018–2020 for females, males, and the total population, separately for adults and the elderly. Percentage changes between the two time periods are indicated above each group.

The results of the WISH2.0 calculation are shown in Figure 3. On average, the elderly scored 74 out of 150 in 2005–2006 and 76 in 2018–2020 (respectively, corresponding to 49 and 51% of the theoretical maximum). Adults scored 72 and 69, respectively, in the two assessed dietary surveys (respectively, corresponding to 48 and 46% of the theoretical maximum). This reflects a 2.8% increase among the elderly and a 5.1% decrease among adults. Within the adult group, females had higher scores than males in both survey periods, while among the elderly, scores between genders were similar, with a slight advantage for males in 2018–2020.

**Figure 4:**
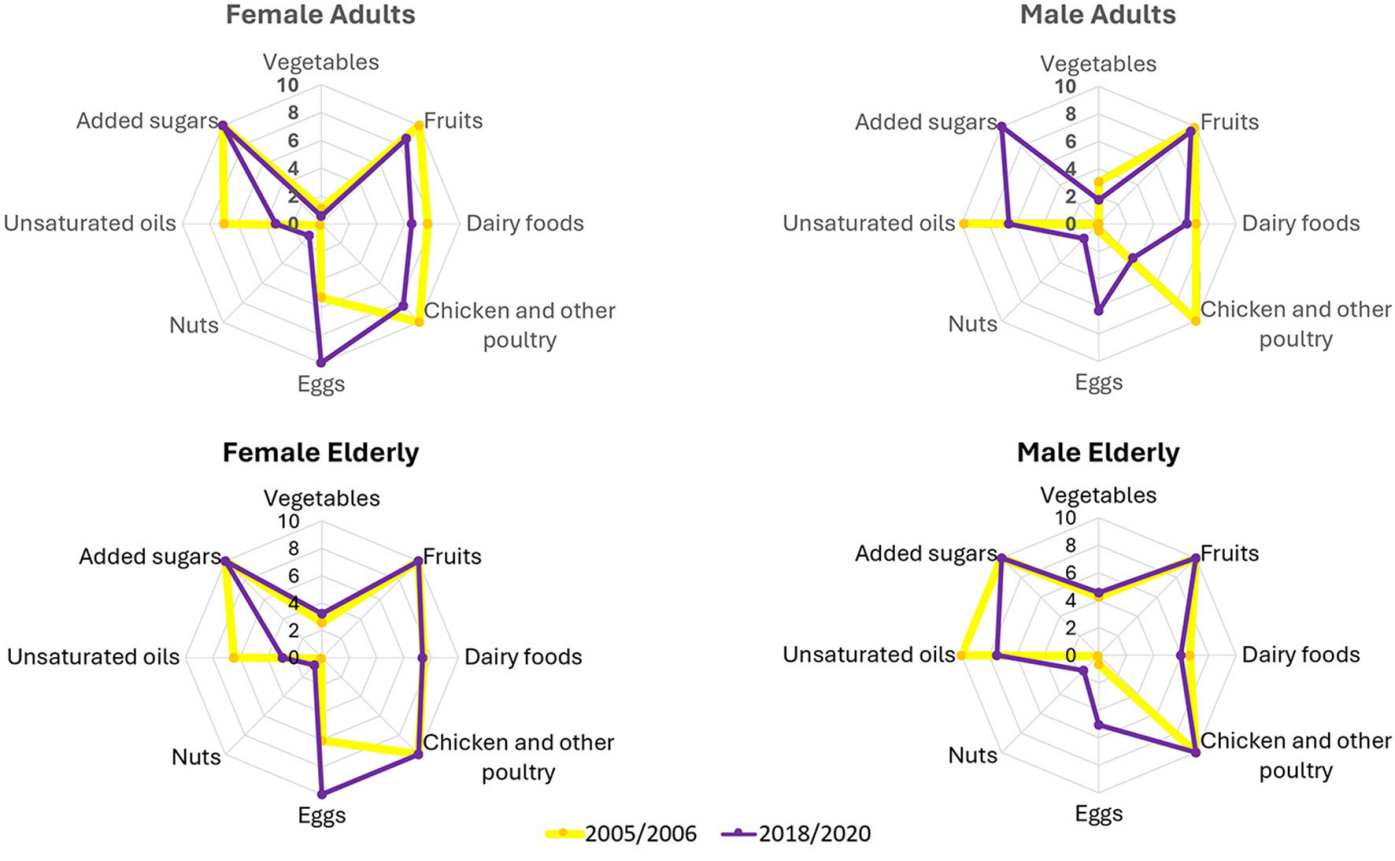
Comparison of WISH component scores between 2005–2006 and 2018–2020 for females and males, separate for adults and the elderly. Each radar graph shows the sub-scores for the food items of each demographic group. Only the food items that exhibited changes over time or differences across demographic groups are included.

As reported in Figure 4 and Supplementary Table S5, the consumption of unsaturated fats in males (adult and elderly) corresponded to the recommendations in 2005–2006, while in females (adult and elderly) the intake resulted lower and not at the recommended levels. For eggs, elderly females met recommendations in 2018–2020, whereas male intakes remained above the recommended threshold despite a decrease. Consumption of processed meat, red meat, alcoholic beverages, and whole grains remained below recommended levels, with all groups scoring the minimum. Nut intake also remained near zero across all groups. In contrast, fish, saturated fats, and added sugar were generally within the recommended range, with the exception of added sugar in adult males in 2005–2006. Fruit, poultry, and dairy products were close to recommended levels across groups.

Among adults, male scores increased from 62 to 67 (+8.1%), while female score dropped from 73 to 70 (−3.6%; Figure 3). This was associated with multiple food group changes (Figure 4 top panels). Egg consumption decreased in both genders, resulting in a 5-point increase (females from 5.3 to 10; males from 0.5 to 6.3). A reduction in added sugar intake contributed to a 10-point gain for males. In contrast, unsaturated fat intake declined (females: from 7 to 3.3; males: from 9.8 to 6.6), as did consumption of chicken and other poultry (females: from 10 to 8.4; males: from 10 to 3.5). Small decreases in vegetable, fruit, and dairy intake contributed to a modest decline in both males (−2 points) and females (−3 points). Nut consumption increased slightly for both genders.

Among the elderly, WISH2.0 scores increased from 73.5 to 76.4 (+4%) in males and from 74.1 to 75.5 (+1.9%) in females (Figure 3). A decrease in egg consumption was associated with a 4-point gain in both males (from 0.7 to 5.1) and females (from 6.1 to 10). Unsaturated fat consumption declined in both sexes, with a 2.6 point loss in males (from 10 to 7.4) and a 3.6 point loss in females (from 6.5 to 2.9). Nut intake increased slightly in both groups. No other major changes were observed in the remaining food categories (Figure 4 down panels).

Sub-score results are presented in Table 2. Most sub-scores were close to 50% of their respective theoretical maximum values, with the exception of the sub-score for adherence to recommendations on low consumption of unhealthy foods, which reached only 40% of the maximum. The sub-score related to high consumption of low-impact foods decreased across all groups between 2005–2006 and 2018–2020, except for adult males, for whom it increased. The sub-score for high consumption of high-impact foods increased in all population groups. When considering both environmental sub-scores, it can be considered that environmental impact worsened among adults and females, while a slight improvement was observed among the elderly and males. The sub-score measuring adherence to recommendations for high consumption of healthy foods declined among adults but improved among the elderly. In both survey periods, the elderly had higher scores than adults for all sub-scores. The gap between the two age groups widened over time.

**Table 2:** World Index for Sustainability and Health (WISH2.0) sub-scores (high consumption of healthy foods; low consumption of unhealthy foods; high, consumption of low-impacting foods; low consumption of high-impacting foods): temporal trends by sex and age group (adults and elderly).

**Year**	**Population group**	**Gender**	**Total WISH2.0**	**WISH2.0 sub-scores**			
				**High consumption of healthy foods**	**Low consumption of unhealthy foods**	**High consumption of low-impacting foods**	**Low consumption of high-impacting foods**
Theoretical maximum achievable				100	50	60	90
2005–2006	Adults	Female	72.7	52.7	20.0	29.6	43.1
		Male	62.0	52.0	10.0	24.3	37.7
		Total	72.4	52.4	20.0	32.0	40.4
	Elderly	Female	74.1	54.1	20.0	30.5	43.6
		Male	73.5	53.5	20.0	36.1	37.4
		Total	74.0	54.0	20.0	33.5	40.5
2018–2020	Adults	Female	70.1	50.1	20.0	24.0	46.1
		Male	67.1	47.1	20.0	29.3	37.8
		Total	68.7	48.7	20.0	26.6	42.1
	Elderly	Female	75.5	55.5	20.0	27.3	48.1
		Male	76.4	56.4	20.0	33.8	42.6
		Total	76.0	56.0	20.0	30.6	45.5

The theoretical maximum score achievable for each sub-score is shown in the first row.

### 3.4 The ultra-processed food consumption in Italy

Analyzing food consumption in Italy based on the NOVA classification (Table 3), it could be highlighted that unprocessed or minimally processed foods (NOVA1) were predominant, accounting for ~80% of total consumption by weight. In contrast, UPFs (NOVA4) contributed only slightly more than 5%.

**Table 3:** Food consumption in Italy categorized according to the four NOVA categories: temporal trends in absolute intake quantity (g) and proportion (%), by sex and age group (adults and elderly).

**Year**	**Group**	**Gender**	**Unprocessed or minimally processed foods (NOVA 1)**		**Processed culinary ingredients (NOVA 2)**		**Processed foods (NOVA 3)**		**Ultra-processed foods (NOVA 4)**	
2005–2006	Adults	Female	1,636	78.8%	64	3.1%	258	12.4%	119	5.7%
		Male	1,621	71.3%	77	3.4%	432	19.0%	145	6.4%
		Total	1,629	74.9%	71	3.2%	345	15.8%	132	6.1%
	Elderly	Female	1,654	80.2%	62	3.0%	263	12.7%	85	4.1%
		Male	1,654	73.1%	72	3.2%	448	19.8%	88	3.9%
		Total	1,654	76.5%	67	3.1%	355	16.4%	86	4.0%
2018–2020	Adults	Female	2,227	83.2%	40	1.5%	215	8.0%	196	7.3%
		Male	2,390	77.7%	48	1.5%	384	12.5%	254	8.3%
		Total	2,309	80.3%	44	1.5%	299	10.4%	225	7.8%
	Elderly	Female	2,293	85.2%	38	1.4%	227	8.4%	134	5.0%
		Male	2,459	80.7%	48	1.6%	430	14.1%	109	3.6%
		Total	2,376	82.8%	43	1.5%	329	11.5%	122	4.2%

Regarding gender differences, females tended to consume more unprocessed or minimally processed foods (NOVA1: 82% on average vs. 76% for males), whereas males consumed more PFs (NOVA3: 16% on average vs. 10% for females). No relevant gender differences were observed in the consumption of processed culinary ingredients (NOVA2) or UPFs (NOVA4). UPFs were consumed more by adults (around 7%) than by the elderly (~4%).

Examining changes over the 15-year period, an increase in the consumption of unprocessed or minimally processed foods was observed (rising from ~76 to 82%). However, this was also accompanied by a small increase in UPF consumption (from ~5 to 6%). The increase in unprocessed or minimally processed foods was more pronounced in males, whereas the rise in UPF consumption was greater among adults. In contrast, a decline was observed in the consumption of processed culinary ingredients (from ~3 to 1.5%) and PFs (from ~16 to 11%). While the reduction in processed culinary ingredients was consistent across all groups, the decrease in PFs consumption was greater among women and adults. Interestingly, although adults exhibited the largest increase in UPFs, their overall intake of PFs and UPFs declined as a percentage of total consumption (from ~22 to 18%), even though the absolute consumed quantity in weight increased.

### 3.5 The proportion of energy, nutrients, and alcohol derived from ultra-processed foods in Italy

The nutrient composition of foods consumed in Italy during the two analyzed periods, categorized according to the four NOVA groups, is presented in Table 4. In both periods, most of the foods consumed in Italy belonged to the unprocessed or minimally processed category. In 2005–2006, this group accounted for 37% of total energy intake and 76% of total consumed quantities, while in 2018–2020, it contributed 35% of total energy and 81% of total food weight. However, the consumption of UPFs increased over time, contributing to 12% of total energy intake in 2005–2006 and rising to 23% in 2018–2020. This increase had a notable impact on nutrient intake over the 15-year period. The proportion of sugars derived from UPFs rose from 18 to 28%, while the share of animal fats from UPFs increased from 17 to 29%. Similarly, the contribution of saturated fats from UPFs grew from 16 to 29%. Conversely, the proportion of vegetable proteins provided by UPFs increased from 11% in 2005–2006 to 23% in 2018–2020, and the fiber contribution from UPFs rose from 6 to 13% over the same period. Despite these shifts, the overall nutrient profile of UPFs consumed in Italy remained relatively stable being primarily composed of carbohydrates (34%), sugars (13%), and fats (10%).

**Table 4:** Energy, nutrients, and alcohol intake from the four NOVA groups in Italy: temporal trends in absolute average daily intake (g, kcal) and proportional contribution (%), in the overall population.

**Year**	**2005–2006**				**2018–2020**			
**NOVA group**	**1**	**2**	**3**	**4**	**1**	**2**	**3**	**4**
**The average intake**								
Consumed quantity	1,645	69	351	114	2,366	44	318	188
Energy (calories)	793	479	631	266	717	312	552	474
Carbohydrate	127	18	66	36	94	8	57	68
Sugar	38	18	9	14	44	8	10	24
Fat	13	46	20	11	18	31	17	19
Animal fat	9	9	16	7	10	1	14	10
Vegetable fat	4	37	0.9	4	8	30	1	7
Saturated fat	4	10	9	5	4	6	8	7
Protein	48	0.5	30	6	48	0.1	26	11
Animal protein	30	0.3	18	3	32	0.1	17	5
Vegetable protein	18	0.2	5	3	15	0.1	4	6
Fiber	13	0.2	5	1	12	0.1	4	3
Alcohol	0.4	0.0	11	1	0.4	0.0	10	0.8
**Proportion among the four NOVA groups (row percentage)**								
Consumed quantity	76%	3%	16%	5%	81%	1.5%	11%	6%
Energy (calories)	37%	22%	29%	12%	35%	15%	27%	23%
Carbohydrate	51%	7%	27%	15%	42%	4%	25%	30%
Sugar	48%	23%	12%	18%	51%	9%	11%	28%
Fat	15%	51%	22%	13%	21%	36%	20%	22%
Animal fat	23%	21%	39%	17%	28%	3%	40%	29%
Vegetable fat	8%	82%	2%	8%	18%	65%	2%	14%
Saturated fat	16%	36%	31%	16%	18%	22%	31%	29%
Protein	57%	0.6%	35%	8%	56%	0.1%	31%	13%
Animal protein	59%	0.6%	35%	6%	60%	0.1%	31%	9%
Vegetable protein	70%	0.7%	19%	11%	60%	0.2%	17%	23%
Fiber	67%	0.8%	26%	6%	64%	0.3%	22%	13%
Alcohol	3%	0.0%	92%	5%	3%	0.0%	89%	7%
**Nutrient intake proportion within each NOVA group (column percentage)**								
Consumed quantity	100%	100%	100%	100%	100%	100%	100%	100%
Carbohydrate	8%	26%	19%	32%	4%	18%	18%	36%
Sugars	2%	26%	3%	13%	2%	18%	3%	13%
Fat	0.8%	66%	6%	10%	0.8%	72%	5%	10%
Animal fat	0.6%	13%	5%	6%	0.4%	2%	4%	5%
Vegetable fat	0.2%	54%	0.2%	3%	0.4%	69%	0.4%	4%
Saturated fat	0.3%	15%	3%	4%	0.2%	13%	2%	4%
Protein	3%	0.7%	8%	6%	2%	0.3%	8%	6%
Animal protein	2%	0.5%	5%	3%	1.4%	0.1%	5%	2%
Vegetable protein	1.1%	0.2%	1.3%	2%	0.6%	0.1%	1.3%	3%
Fiber	0.8%	0.2%	1.4%	1.1%	0.5%	0.1%	1.3%	1.4%
Alcohol	0.0%	0.0%	3%	0.5%	0.0%	0.0%	3%	0.4%

In 2005–2006, the proportion of energy intake from UPFs was similar between the sexes, accounting for 12% in males and 13% in females (Supplementary Table S6). However, by 2018–2020, females showed a slightly higher energy intake from UPFs (24%) compared to males (21%). This increase was primarily driven by a rise in the proportion of carbohydrates derived from UPFs, which increased from 15 to 32% in females and from 13 to 30% in males. In both sexes, the proportion of sugar intake from UPFs also increased, reaching comparable levels over time (2005–2006: 17% in females and 18% in males; 2018–2020: 27% in females and 28% in males). A similar pattern was observed for animal fats with both sexes increasing from 17% in 2005–2006 to 29% in 2018–2020. Saturated fat intake from UPFs also rose in both groups from 16% in 2005–2006 to 29% in females and 27% in males in 2018–2020. In addition, the proportion of vegetable proteins derived from UPFs increased in both sexes—from 11% in females and 10% in males in 2005–2006 to 24 and 23%, respectively, in 2018–2020. A similar trend was observed for fiber intake, which rose from 6% in both sexes in 2005–2006 to 14% in females and 12% in males in 2018–2020. Overall, the upward trends in UPF-derived nutrient intake were consistent across both genders.

Among adults, the proportion of energy intake from UPFs was consistently higher than that of the elderly in both periods: 14 vs. 11% in 2005–2006 and 26 vs. 20% in 2018–2020 (Supplementary Table S7). The increase observed over time was largely attributed to a higher intake of specific nutrients from UPFs, particularly animal fats (rising from 18 to 31% in adults and from 16 to 27% in the elderly), saturated fats (from 18 to 31% in adults and from 15 to 26% in the elderly), and sugars (from 21 to 37% in adults and from 15 to 21% in the elderly). The contribution of vegetable proteins from UPFs also increased from 12 to 24% in adults and from 10 to 22% in the elderly. Overall, the increases in UPF-derived nutrient intake were more pronounced in adults compared to the elderly. Notable differences were also observed in the rise of fiber intake (from 7 to 16% in adults and from 6 to 11% in the elderly) and alcohol intake from UPFs (from 7 to 12% in adults and from 2 to 4% in the elderly), further contributing to the widening nutritional gap between the two age groups over time.

In Italy, alcohol intake primarily came from wine and beer, which are classified as processed foods (NOVA 3). These beverages accounted for 92% of total alcohol consumption in 2005–2006, decreasing slightly to 89% in 2018–2020 (Supplementary Table S4). This decline was offset by an increase in the consumption of distilled alcoholic drinks, classified as UPFs (NOVA 4), which rose from 5% in 2005–2006 to 7% in 2018–2020 (Supplementary Table S4). The rise in alcohol intake from distilled drinks was particularly noticeable among males, increasing from 5% in 2005–2006 to 7% in 2018–2020, while it remained stable at 4% among females (Supplementary Table S6). Across age groups, the consumption of distilled alcoholic beverages increased more significantly among adults, rising from 7% in 2005–2006 to 12% in 2018–2020, whereas in the elderly, the increase was more moderate, from 2 to 4% over the same period (Supplementary Table S7).

## 4 Discussion

This study provides comprehensive insights into the evolution of dietary patterns in Italy between 2005–2006 and 2018–2020, comparing dietary habits with Italian Healthy Eating Guidelines (33) and EAT-Lancet recommendations for a healthy and sustainable diet (1). In addition, the consumption of UPFs was also examined as an indicator of Westernization of the diet (41). Several key findings emerge from this analysis that provide an understanding of the evolving trends in diet quality and their potential implications for public health and sustainability.

The Italian diet remained characterized by high consumption of traditional Mediterranean foods such as fruits, vegetables, and cereals like bread, pasta, and rice which were the most consumed food groups.

Over the 15-year period, dietary patterns in Italy revealed significant shifts shaped by age and gender. Adults showed declining adherence to dietary recommendations (AIDGI and WISH2.0), particularly among males, while elderly individuals—especially females—demonstrated improved or more stable scores, suggesting greater dietary resilience. Notable trends included reduced intake of staple carbohydrates and nutrient-dense foods, alongside increased consumption of discretionary items such as salty snacks, cakes, and sugary drinks—particularly in elderly males. Persistent gender gaps, such as higher red meat and alcohol consumption in males and more health-conscious behaviors in females, highlight the need for gender-sensitive interventions. Despite the acknowledged methodological differences between the dietary surveys conducted in 2005–2006 and 2018–2020, the EFSA Comprehensive Database remains the only available resource for analyzing long-term dietary trends at the national level (42). These differences introduce some degree of variability that may affect the comparability of results over time. It is, therefore important to consider that part of the variation could stem from these methodological changes. Although differences in data collection methods must be considered, the consistency in sampling strategy and population coverage supports the reliability of observed trends, especially when focusing on relative rather than absolute changes evaluated with dietary quality indices. Moreover, the direction of dietary shifts observed, such as the decline in fruit and vegetable consumption, and the rise in the UPF intake, has been corroborated by other studies conducted in Italy (21, 41), further reinforcing the plausibility of the trends identified through the EFSA Comprehensive Database analysis. The diet quality indices used in this study, namely, AIDGI and WISH2.0, showed scores close to 50% of the theoretical maximum achievable indicating that there is substantial room for improvement in dietary quality among the study population. This suggests that while some aspects of the healthy eating pattern are being followed, there are notable gaps that may need to be addressed to enhance overall diet quality and alignment with recommended nutritional guidelines.

In overall terms, the elderly and women showed better eating habits, both when comparing with the EAT-Lancet recommendations (WISH2.0) and the Italian Healthy Eating Guidelines (AIDGI). The greatest difference was observed between adult and elderly males. These findings were in line with those reported by Dinu et al. (43), who showed greater adherence to dietary recommendations in females and the elderly than in males and adults.

The results related to food groups showed that animal source food intake was generally excessive, while vegetable source food consumption was far lower than the recommendations. Overall, across both genders and age groups, the most problematic food categories were alcohol and processed meats, which consistently received a score of zero in both indices. Red meat also had low scores, with non-zero values appearing only in AIDGI for women, while legumes scored zero in AIDGI and remained very low in WISH2.0, sugar-sweetened beverages (for AIDGI) and whole grains (for WISH2.0) consistently received zero scores. This dietary pattern reflects the ongoing Westernization of the Italian diet, which should be considered in terms of its potential effects on health and the environment in terms of sustainability (44).

Notably, nuts showed very low scores in WISH2.0 but had the highest scores in AIDGI in 2018–2020. This discrepancy was due to differing recommendations for this food group: The Italian Healthy Eating Guidelines suggested 30 g 2.5 times a week, while the EAT-Lancet recommendations advised 50 g/day.

The maintenance of traditional Mediterranean characteristics of the Italian dietary pattern was demonstrated by the adherence to recommendations for foods such as saturated fats, potatoes, poultry, fish, and eggs, with scores close to the maximum, as well as fruits, which scored high in WISH2.0 and medium in AIDGI. This discrepancy, such as that observed with nuts, could be explained by the fact that the Italian Food-based Dietary Guidelines recommend a larger amount of fruit compared to the EAT-Lancet recommendations (450 vs. 200 g/day).

Temporal trends had different characteristics in adults and the elderly. Both AIDGI and WISH2.0 scores indicated a more favorable change over time in the elderly compared to adults (AIDGI: +5.6% in the elderly, −5.9% in adults; WISH2.0: +2.8% in the elderly, and −5.1% in adults). The general decline in adherence to the Italian Dietary Guidelines of Italian adults confirmed the hypothesis of a progressive departure from the Mediterranean Diet especially for the youngest. This trend aligns with previous studies that have reported similar dietary shifts in other Southern European countries (11). The decline in vegetable and fresh fruit consumption in adults is concerning given the well-documented health benefits of these food groups (45). This decline is particularly relevant given the extensive national educational and promotional campaigns aimed at increasing vegetable consumption. It suggests that public health interventions in Italy have yet to make a significant impact on dietary behaviors, especially among younger age groups. However, the elderly population demonstrated relatively higher adherence to dietary recommendations, suggesting that traditional eating habits may be more resilient among older age groups.

The use and adaptation of WISH2.0 in this study allowed for a more comprehensive evaluation of diet quality in Italy by integrating sustainability considerations. The findings indicate that adherence to sustainable dietary recommendations remains suboptimal, reinforcing concerns about the environmental impact of dietary patterns (46). The decline in the consumption of recommended food groups and the concurrent rise in foods to be limited or avoided confirmed the opportunity to emphasize not only nutritional adequacy but also sustainability in the Italian dietary guidelines (47). The findings of this study underscore the need for integrated approaches that address both health and environmental concerns. The inclusion of additional food groups in WISH2.0, such as processed meat and alcohol, provides a more nuanced understanding of how dietary habits influence both human and planetary health. The changes in alcohol consumption patterns warrant particular attention, especially the increased intake of distilled alcoholic beverages. This shift may reflect broader changes in drinking behaviors, potentially influenced by cultural and economic factors (48). The greater rise in distilled alcohol consumption among adults compared to the elderly suggests that younger individuals are adopting different drinking patterns, which could have long-term public health implications given the known associations between alcohol consumption and chronic disease risk (49).

The NOVA classification of Italian food consumption confirmed that the intake of UPFs in Italy was lower than in other European countries (20). The alignment of the present results with those from Mertens et al. (20) is not unexpected, as both studies used the EFSA comprehensive database for their estimates, and the NOVA classification followed the same methodology. Similarly, the study by Ruggiero et al. (21) reached the same conclusion, indicating that UPFs contribute only a modest proportion of energy to the diets of the analyzed cohort. Thus, the overall intake of UPFs in Italy appears to be relatively moderate, potentially due to the persistence of traditional dietary patterns, such as the Mediterranean diet, which emphasizes fresh and minimally processed foods (50). In support of these findings were the results of the EPIC cohort study, on the consumption of UPFs and risk of multimorbidity of cancer and cardiometabolic diseases, which showed that Italy and Spain are among the European countries with the lowest consumption of UPF and high adherence to DM (51). However, the present study revealed a slight increase in UPF consumption in Italy over time, rising from 5% in 2005–2006 to 6% in 2018–2020. This upward trend should not be overlooked, particularly among adults, where the increase was twice as high, reaching 8% in 2018–2020. This finding supports the hypothesis that UPF consumption has risen in recent years, aligning with global trends of increased reliance on UPFs (52). The authors' interpretation of these findings is that, while UPF consumption in Italy is increasing, it may not yet be at levels high enough to significantly deteriorate overall diet quality. However, it should be precise that the target groups of this study were adults and elderly; other groups of the population, such as children and adolescents, or people particularly vulnerable such as those low socio-economic status consumers, could be more exposed to high levels of consumption of UPFs that could contribute to poorer dietary quality (53).

In both surveys and across all age groups, females demonstrated greater attention to their diet than males, consuming more unprocessed or minimally processed foods (NOVA 1). This finding reinforces the overall higher dietary quality observed in females compared to males.

The nutritional breakdown by NOVA categories revealed that, although ultra-processed foods (NOVA 4) accounted for only 6% of total food consumption by weight in 2018–2020, they contributed a substantial 23% of total energy intake. This reflects a significant increase in the contribution of ultra-processed foods to overall energy intake during the most recent assessment period. The increase in the proportion of sugars, animal fats, and saturated fats from UPFs is particularly concerning, given their well-documented links to adverse health outcomes (53, 54). However, UPFs could be also a source of vegetable proteins and fiber that increased through the years, and this could be related to the increased availability on the market of UPFs coming from a vegetable matrix (e.g., legumes) used as a substitute for meat (e.g., veggie burgers) (55). The present data would not permit to say if the proportion of vegetable proteins and fiber compensate for the negative effects of increased intake of sugars and saturated fats; however, a reflection on the nutritional profile of UPFs and the different impact of their nutritional composition should be carried out. Moreover, the age-related differences in UPF consumption, with higher intake among adults compared to the elderly, are consistent with the findings from other studies (56), which suggest that younger populations are more exposed to processed food environments and marketing strategies promoting UPFs (57).

The quantification of UPFs and their nutrient composition in Italy presents challenges in determining whether the concept of UPFs should be incorporated into the Italian Dietary Guidelines. As highlighted by Scrinis and Monteiro (58), the UPF category includes a wide variety of products with diverse compositional and processing characteristics, some of which may have a more favorable nutrient or ingredient profile than others. These differences, along with consumption patterns, may contribute to varying health effects across different UPF subgroups. According to Cordova et al. (51), the consumption of sugar-sweetened and artificially sweetened beverages, as well as animal-based UPFs, has been linked to an increased risk of multimorbidity. In contrast, ultra-processed breads and cereals, and plant-based meat alternatives have been associated with a lower risk. Given the national consumption patterns of UPFs in Italy, the dietary guidelines may benefit from prioritizing nutritional quality and nutritional profile over the degree of processing. Although UPFs represent a relatively low percentage of total intake in Italy, their upward trend and association with lower food quality suggest the importance of preventive action. However, it is essential to acknowledge that not all nutritionally unbalanced foods fall into the UPFs category. Therefore, focusing solely on reducing UPFs intake is not sufficient. For example, sugar, salt, and meat (especially red meat) contribute to dietary excesses regardless of their NOVA classification and should be considered in public health strategies alongside the reduction of UPFs.

This study has both strengths and limitations. The most important strength is related to its novelty and key findings. To the best of the authors' knowledge, this is the first time that official Italian food consumption data from the EFSA Comprehensive Database have been analyzed for temporal trends, providing comparative insights into changes over the 15-year gap between the two data collection periods. In addition, the evaluation of the two diet quality indicators, namely, WISH2.0 and AIDGI, introduces innovative aspects for conceptual and methodological reasons. First, the implementation of WISH2.0, which incorporates sustainability into diet quality assessment, aligns with the growing need for a more holistic evaluation that integrates both nutritional value and sustainability aspects. In methodological terms, the original AIDGI scoring system was adapted to fit the available dataset. By converting qualitative frequency scales into quantitative measurements, the study enhanced the precision of the data, thereby improving the accuracy of adherence assessment. Notably, the subdivision of the “more than once a day” category into two separate groups allowed for a more precise reflection of dietary recommendations for food consumed daily. This modification strengthened the indicator's ability to differentiate between varying levels of adherence, minimizing potential distortions associated with broader qualitative scales. Furthermore, this refinement facilitates comparisons between datasets that use different standard portion sizes, ensuring that frequency data remain comparable across studies. This enhanced methodology could serve as a model for future dietary assessment studies. However, although the assessment approach preserved the original AIDGI structure and was based on official dietary recommendations, further validation, such as sensitivity analyses or comparison with health outcomes, is recommended to assess the robustness of the adapted system.

The use of pooled data represents a key limitation of this study in consideration that the raw data at the individual level would allow for a more detailed and accurate analysis of dietary patterns, capturing individual variations that might be lost when relying on average. This is particularly relevant for food groups, such as red and processed meat, alcohol, and legumes, where small differences in consumption could be lost when the average data are synthesized in the calculation of the indicators. However, at the population level, given that the consumption of these food groups deviates significantly from recommendations, the use of pooled data is unlikely to substantially affect the overall findings and conclusions provided on the average Italian population groups. Another limitation of this study is the absence of stratification by socioeconomic status, such as income and education, which are well-established determinants of diet quality (59). Given the substantial body of literature on this topic (60), it would be valuable to explore these dimensions in future analyses of the databases used in this study. A further shortcoming of this study was that the FoodEx2 coding system did not allow for a distinction between home-prepared and industrially produced foods, which could result in the misclassification of certain foods and a potential underestimation of UPFs intake. This is particularly relevant for composite dishes or generic food categories where the preparation method (e.g., homemade vs. packaged) significantly influences the processing level. To mitigate this limitation and enhance the accuracy of UPF classification, the study adopted an approach previously employed in other validated investigations (20, 40) applying specific assumptions and standardized criteria to categorize foods based on available descriptors. While this methodology improves cross-study comparability and allows for more consistent estimates of UPF consumption, it still relies partly on assumptions in the absence of detailed product-level information, underscoring the need for improved data granularity in future dietary assessments.

## 5 Conclusion

This study provides a comprehensive assessment of the quality of Italian dietary patterns, identifying key areas of concern and opportunities for targeted intervention. While the average Italian diet did not drastically deviate from national dietary recommendations, the observed temporal trend suggested a gradual decline in overall diet quality, a rise in UPF consumption, and notable demographic disparities, particularly among adult males. To strengthen public health impact, future research should prioritize longitudinal studies that assess the long-term health consequences of these dietary shifts and evaluate the effectiveness of concrete policy measures. In particular, interventions such as front-of-pack nutrition labeling, a stricter regulation on UPF advertising (especially to children), and public procurement policies favoring minimally processed, sustainable foods could support healthier food choices. Efforts should also focus on increasing adherence to both the Italian Healthy Eating Guidelines and the EAT-Lancet recommendations by promoting greater intake of whole grains, legumes, fruits, and vegetables and reducing the consumption of red and processed meats, alcohol, and sugary beverages. These findings provide a solid foundation for shaping evidence-based nutrition policies and public education campaigns aimed at improving population health and lowering the environmental impact of current dietary practices. In addition, this study raises important methodological considerations. The adaptation of the AIDGI to food consumption data expressed in grams per day represents an innovative step toward more precise dietary assessment. Future study should continue to refine these tools to support more effective monitoring, evaluation, and policymaking in Italy and across other Mediterranean Countries.

## Data Availability

The raw data supporting the conclusions of this article will be made available by the authors, without undue reservation.
