# Supplementary material for: The 15-year trend in adherence to dietary recommendations and ultra-processed food consumption in Italy
Source: Front Nutr. 2025 Aug 5;12:1623827. doi: 10.3389/fnut.2025.1623827 (PMC12360916; doi:10.3389/fnut.2025.1623827)
Supplement: Supplementary file 1 [file Table_1.docx]

Supplementary Material

**Table S1** Original AIDGI scoring system that for each food group assigns 2 points for full adherence to the dietary guidelines, 1 point for partial adherence, and 0 points for non-adherence.

| **Food groups** | **More than once per day** | **One per day** | **Few times per week** | **Less than once a week** | **Never** |
| --- | --- | --- | --- | --- | --- |
| 1. Bread, pasta, rice | 2 | 1 | 1 |  |  |
| 2. Potatoes |  | 1 | 2 | 2 | 2 |
| 3. Vegetables | 2 | 1 |  |  |  |
| 4. Fresh fruits | 2 | 1 |  |  |  |
| 5. Dairy products |  | 1 | 2 | 1 | 1 |
| 6. Milk and yogurt | 2 | 1 |  |  |  |
| 7. Red meat |  |  | 1 | 2 | 2 |
| 8. Fish and fisheries products |  | 1 | 2 | 1 |  |
| 9. Eggs |  | 1 | 2 | 1 | 1 |
| 10. Poultry |  |  | 2 | 1 | 1 |
| 11.Legumes |  | 1 | 2 |  |  |
| 12. Nuts |  | 1 | 2 | 1 | 1 |
| 13. Cakes and sweet snacks |  | 1 | 2 | 2 | 2 |
| 14. Salty snacks |  |  |  | 1 | 2 |
| 15. Sugary drinks |  |  |  |  | 2 |
| 16. Beer and wine |  |  |  |  | 2 |
| 17. Other alcoholic beverages |  |  |  |  | 2 |
| 18. Processed and cured meet |  |  |  | 1 | 2 |

**Table S2** Benchmark for the revised AIDGI scoring system. The first columns report the recommended portion sizes (in grams) and the recommended daily intake (in grams) for each food group, according to the Italian Dietary Guidelines. The first row presents the conversion of original categorical consumption frequencies (e.g., “More than two per day”) into weekly portion counts. The right side of the table shows the benchmarks of intake ranges (expressed in grams per day). Dark gray cell indicates full adherence to dietary guidelines (score = 2); light gray cell indicates partial adherence (score = 1); white cell indicates non-adherence (score = 0).

| Food groups | Portion sizes | Recommendation per day | More than two per day | | More than once per day | | One per day | | Few times per week | | Less than once a week | | Never |
| --- | --- | --- | --- | --- | --- | --- | --- | --- | --- | --- | --- | --- | --- |
| number of portions per week🡪 |  |  |  | 17.5 | 17.5 | 9 | 9 | 4 | 4 | 1 | 1 | 0 | 0 |
| 1. Bread, pasta, rice | 56.7 | 307.9 | ∞ | 141.8 | 141.8 | 72.9 | 72.9 | 32.4 | 32.4 | 8.1 | 8.1 | 0.0 | 0.0 |
| 2. Potatos | 200 | 57.1 |  |  |  | 257.1 | 257.1 | 114.3 | 114.3 | 28.6 | 28.6 | 0.0 | 0.0 |
| 3. Vegetables | 140 | 350 | ∞ | 350.0 | 350.0 | 180.0 | 180.0 | 80.0 | 80.0 | 20.0 | 20.0 | 0.0 | 0.0 |
| 4. Fresh fruits | 150 | 450 | ∞ | 375.0 | 375.0 | 192.9 | 192.9 | 85.7 | 85.7 | 21.4 | 21.4 | 0.0 | 0.0 |
| 5. Dairy products | 75 | 32.1 |  |  |  | 96.4 | 96.4 | 42.9 | 42.9 | 10.7 | 10.7 | 0.0 | 0.0 |
| 6. Milk and yogurt | 125 | 375 | ∞ | 312.5 | 312.5 | 160.7 | 160.7 | 71.4 | 71.4 | 17.9 | 17.9 | 0.0 | 0.0 |
| 7. Red meat | 100 | 14.3 |  |  |  | 128.6 | 128.6 | 57.1 | 57.1 | 14.3 | 14.3 | 0.0 | 0.0 |
| 8. Fish and fisheries products | 116.7 | 50 |  |  |  | 150.0 | 150.0 | 66.7 | 66.7 | 16.7 | 16.7 | 0.0 | 0.0 |
| 9. Eggs | 50 | 21.4 |  |  |  | 64.3 | 64.3 | 28.6 | 28.6 | 7.1 | 7.1 | 0.0 | 0.0 |
| 10. Poultry | 100 | 28.6 |  |  |  | 128.6 | 128.6 | 57.1 | 57.1 | 14.3 | 14.3 | 0.0 | 0.0 |
| 11.Legumes | 100 | 42.9 |  |  |  | 128.6 | 128.6 | 57.1 | 57.1 | 14.3 | 14.3 | 0.0 | 0.0 |
| 12. Nuts | 30 | 8.6 |  |  |  | 38.6 | 38.6 | 17.1 | 17.1 | 4.3 | 4.3 | 0.0 | 0.0 |
| 13. Cakes and sweet snacks | 52 | 18.6 |  |  |  | 66.9 | 66.9 | 29.7 | 29.7 | 7.4 | 7.4 | 0.0 | 0.0 |
| 14. Salty snacks | 30 | 2.1 |  |  |  | 38.6 | 38.6 | 17.1 | 17.1 | 4.3 | 4.3 | 0.0 | 0.0 |
| 15. Sugary drinks | 250 | 17.9 |  |  |  | 321.4 | 321.4 | 142.9 | 142.9 | 35.7 | 35.7 | 0.0 | 0.0 |
| 16. Beer and wine | 227.5 | 0 |  |  |  | 292.5 | 292.5 | 130.0 | 130.0 | 32.5 | 32.5 | 0.0 | 0.0 |
| 17. Other alcoholic beverages | 57.5 | 0 |  |  |  | 73.9 | 73.9 | 32.9 | 32.9 | 8.2 | 8.2 | 0.0 | 0.0 |
| 18. Processed and cured meet | 50 | 3.6 |  |  |  | 64.3 | 64.3 | 28.6 | 28.6 | 7.1 | 7.1 | 0.0 | 0.0 |

**Table S3** WISH2.0 scoring system. The underlined food groups were those added to the original WISH. The table shows, for each food group, the recommended daily intake (in grams per day) and its categorization according to healthiness and environmental impact. The right side of the table shows the benchmarks of the three intake ranges (expressed in grams per day): full adherence (dark gray cell, score=10), partial adherence (light gray cell, score = between 0 and 10), and non-adherence (white cell, score=0).

| **Food groups** | **Recommended intake in g/day (Lower and Upper Range of intake)** | **Healthiness** | **Environmental impact** | **10 points** | **Ratio between 0 and 10** | **0 points** |
| --- | --- | --- | --- | --- | --- | --- |
| Whole grains | ≥125 (100-150) | Protective | Low | ≥125 | 100-125 | <100 |
| Vegetables | 300 (200-600) | Protective | Low | ≥300 | 200-300 | <200 |
| Fruit | 200 (100-330) | Protective | Low | ≥200 | 100-200 | <100 |
| Dairy foods | 250 (0-500) | Protective | Medium | 250-500 | 0-250 | >500 |
| Red meat | 14 (0-28) | Limit | High | ≤14 | 14-28 | >28 |
| Fish | 28 (0-100) | Protective | High | 28-100 | 0-28 | >100 |
| Eggs | 13 (0-25) | Neutral | Medium | ≤13 | 13-25 | >25 |
| Chicken and other poultry | 29 (0-58) | Neutral | Medium | ≤29 | 29-58 | >58 |
| Legumes | 75 (0-100) | Protective | Low | ≥75 | 0-75 | 0 |
| Nuts | 50 (0-75) | Protective | Medium | 50-75 | 0-50 | >75 |
| Added sugars | 31 (0-31) | Limit | Low | ≤31 |  | >31 |
| Alcoholic beverages | 0 (0-10) | Avoid | High | 0 |  | >0 |
| Processed meat | 2 (0-4) | Limit | High | ≤2 | 2-4 | >4 |
| Saturated oils | 11.8 (0-11.8) | Limit | High | ≤11.8 |  | >11.8 |
| Unsaturated oils | 40 (20-80) | Protective | Low | ≥40 & ≤ 80 | 20-40 | <20 & >80 |

**Table S4** AIDGI observed scores: total scores and food group sub-scores by age, gender and temporal trend.
Food groups highlighted in yellow indicate a change over time (values in bold).

| Population Group | Adults | | | | | | Elderly | | | | | |
| --- | --- | --- | --- | --- | --- | --- | --- | --- | --- | --- | --- | --- |
| Gender | Female | | Male | | Total | | Female | | Male | | Total | |
| Year | 2005 2006 | 2018 2020 | 2005 2006 | 2018 2020 | 2005 2006 | 2018 2020 | 2005 2006 | 2018 2020 | 2005 2006 | 2018 2020 | 2005 2006 | 2018 2020 |
| **AIDGI** | **18** | **17** | **17** | **16** | **17** | **16** | **19** | **19** | **17** | **16** | **18** | **19** |
| Bread, pasta, rice | 2 | **1** | 2 | 2 | 2 | 2 | 2 | 2 | 2 | 2 | 2 | 2 |
| Potatoes | 2 | 2 | 2 | 2 | 2 | 2 | 2 | 2 | 2 | 2 | 2 | 2 |
| Vegetables | 1 | 1 | 1 | 1 | 1 | 1 | 1 | 1 | 1 | 1 | 1 | 1 |
| Fresh fruits | 1 | 1 | 1 | 1 | 1 | 1 | 1 | 1 | 1 | 1 | 1 | 1 |
| Dairy products | 1 | 1 | 1 | 1 | 1 | 1 | 1 | **2** | 1 | 1 | 1 | 1 |
| Milk and yogurt | 1 | 1 | 1 | 1 | 1 | 1 | 1 | 1 | 1 | 1 | 1 | 1 |
| **Red meat** | **1** | **1** | **0** | **0** | **0** | **0** | **1** | **1** | **0** | **0** | **0** | **1** |
| Fish and fisheries products | 2 | 2 | 2 | 2 | 2 | 2 | 2 | 2 | 2 | **1** | 2 | 2 |
| Eggs | 2 | 2 | 2 | 2 | 2 | 2 | 2 | 2 | 2 | 2 | 2 | 2 |
| Poultry | 2 | 2 | 2 | 2 | 2 | 2 | 2 | 2 | 2 | 2 | 2 | 2 |
| Legumes | 0 | 0 | 0 | 0 | 0 | 0 | 0 | 0 | 0 | 0 | 0 | 0 |
| **Nuts** | **1** | **2** | **1** | **2** | **1** | **2** | **1** | **1** | **1** | **2** | **1** | **2** |
| **Cakes and sweet snacks** | **1** | **1** | **1** | **0** | **1** | **0** | **2** | **1** | **1** | **1** | **2** | **1** |
| **Salty snacks** | **1** | **0** | **1** | **0** | **1** | **0** | **1** | **1** | **1** | **0** | **1** | **1** |
| Sugary drinks | 0 | 0 | 0 | 0 | 0 | 0 | 0 | 0 | 0 | 0 | 0 | 0 |
| Beer and wine | 0 | 0 | 0 | 0 | 0 | 0 | 0 | 0 | 0 | 0 | 0 | 0 |
| Other alcoholic beverages | 0 | 0 | 0 | 0 | 0 | 0 | 0 | 0 | 0 | 0 | 0 | 0 |
| Processed and cured meet | 0 | 0 | 0 | 0 | 0 | 0 | 0 | 0 | 0 | 0 | 0 | 0 |

**Table S5** WISH2.0 observed scores: total scores and food group sub-scores by age, gender and temporal trend.
Food groups highlighted in yellow indicate a change over time (values in bold).

| Population Group | Adults | | | | | | Elderly | | | | | |
| --- | --- | --- | --- | --- | --- | --- | --- | --- | --- | --- | --- | --- |
| Gender | Female | | Male | | Total | | Female | | Male | | Total | |
| Year | 2005 2006 | 2018 2020 | 2005 2006 | 2018 2020 | 2005 2006 | 2018 2020 | 2005 2006 | 2018 2020 | 2005 2006 | 2018 2020 | 2005 2006 | 2018 2020 |
| **WISH** | **72.65** | **70.06** | **62.04** | **67.05** | **72.42** | **68.74** | **74.07** | **75.46** | **73.50** | **76.41** | **73.98** | **76.05** |
| Whole grains | 0.0 | 0.0 | 0.0 | 0.0 | 0.0 | 0.0 | 0.0 | 0.0 | 0.0 | 0.0 | 0.0 | 0.0 |
| Vegetables | 1.1 | 0.6 | 3.1 | 1.7 | 2.1 | 1.1 | 2.6 | 3.2 | 4.2 | 4.6 | 3.4 | 3.9 |
| Fruits | 10.0 | 8.7 | 9.9 | 9.5 | 10.0 | 9.1 | 10.0 | 10.0 | 10.0 | 10.0 | 10.0 | 10.0 |
| Dairy foods | 7.7 | 6.5 | 7.1 | 6.4 | 7.4 | 6.5 | 7.5 | 7.4 | 6.6 | 6.0 | 7.1 | 6.7 |
| Red meat | 0.0 | 0.0 | 0.0 | 0.0 | 0.0 | 0.0 | 0.0 | 0.0 | 0.0 | 0.0 | 0.0 | 0.0 |
| Fish | 10.0 | 10.0 | 10.0 | 10.0 | 10.0 | 10.0 | 10.0 | 10.0 | 10.0 | 10.0 | 10.0 | 10.0 |
| **Eggs** | **5.3** | **10.0** | **0.5** | **6.3** | **2.9** | **8.4** | **6.1** | **10.0** | **0.7** | **5.1** | **3.4** | **7.6** |
| **Chicken and other poultry** | **10.0** | **8.4** | **10.0** | **3.5** | **10.0** | **5.9** | 10.0 | 10.0 | 10.0 | 10.0 | 10.0 | 10.0 |
| Legumes | 1.5 | 1.5 | 1.6 | 1.5 | 1.5 | 1.5 | 1.4 | 1.2 | 1.9 | 1.8 | 1.7 | 1.5 |
| Nuts | 0.1 | 1.2 | 0.1 | 1.5 | 0.1 | 1.4 | 0.1 | 0.8 | 0.1 | 1.6 | 0.1 | 1.2 |
| Added sugars | 10.0 | 10.0 | **0.0** | **10.0** | 10.0 | 10.0 | 10.0 | 10.0 | 10.0 | 10.0 | 10.0 | 10.0 |
| Alcoholic beverages | 0.0 | 0.0 | 0.0 | 0.0 | 0.0 | 0.0 | 0.0 | 0.0 | 0.0 | 0.0 | 0.0 | 0.0 |
| Processed meat | 0.0 | 0.0 | 0.0 | 0.0 | 0.0 | 0.0 | 0.0 | 0.0 | 0.0 | 0.0 | 0.0 | 0.0 |
| Saturated oils | 10.0 | 10.0 | 10.0 | 10.0 | 10.0 | 10.0 | 10.0 | 10.0 | 10.0 | 10.0 | 10.0 | 10.0 |
| **Unsaturated oils** | **7.0** | **3.3** | **9.8** | **6.6** | **8.4** | **4.9** | **6.5** | **2.9** | **10.0** | **7.4** | **8.4** | **5.2** |

**Table S6** Energy, nutrients, and alcohol intake from the four NOVA groups in Italy: temporal trends in average daily intake (g, kcal) and proportional contribution (%) by sexes.

| **The average intake of UPFs** | | | | |
| --- | --- | --- | --- | --- |
| Year | 2005/2006 | | 2018/2020 | |
| Gender | Female | Male | Female | Male |
| Consumed quantity | 102 | 116 | 165 | 181 |
| Energy (calories) | 246 | 277 | 425 | 474 |
| Carbohydrate | 34 | 36 | 62 | 66 |
| Sugars | 13 | 14 | 21 | 24 |
| Fat | 10 | 12 | 17 | 19 |
| Animal fat | 6 | 8 | 9 | 11 |
| Vegetable fat | 3 | 4 | 6 | 7 |
| Saturated fat | 4 | 5 | 6 | 7 |
| Protein | 6 | 7 | 10 | 11 |
| Animal protein | 3 | 3 | 4 | 5 |
| Vegetable protein | 3 | 3 | 6 | 6 |
| Fibre | 1.2 | 1.1 | 2 | 2 |
| Alcohol | 0.2 | 0.8 | 0.2 | 1.2 |
| **Proportion of UPFs group** | | | | |
| (row percentage) | | | | |
| Year | 2005/2006 | | 2018/2020 | |
| Gender | Female | Male | Female | Male |
| Consumed quantity | 5% | 5% | 6% | 6% |
| energy (calories) | 13% | 12% | 24% | 23% |
| Carbohydrate | 15% | 13% | 32% | 30% |
| Sugar | 17% | 18% | 27% | 28% |
| Fat | 12% | 13% | 22% | 22% |
| Animal fat | 17% | 17% | 29% | 29% |
| Vegetable fat | 8% | 8% | 15% | 14% |
| Saturated fat | 16% | 16% | 29% | 29% |
| Protein | 8% | 7% | 14% | 13% |
| Animal protein | 6% | 6% | 9% | 9% |
| Vegetable protein | 11% | 10% | 24% | 23% |
| Fibre | 6% | 6% | 14% | 13% |
| Alcohol | 4% | 5% | 4% | 7% |

**Table S7** Energy, nutrients, and alcohol intake from the four NOVA groups in Italy: temporal trends in average daily intake (g, kcal) and proportional contribution (%) by population groups.

| **The average intake of UPFs** | | | | |
| --- | --- | --- | --- | --- |
| Year | 2005/2006 | | 2018/2020 | |
| Population Group | Adults | Elderly | Adults | Elderly |
| Consumed quantity | 137 | 90 | 232 | 145 |
| Energy (calories) | 302 | 231 | 539 | 408 |
| Carbohydrate | 40 | 32 | 78 | 58 |
| Sugar | 17 | 12 | 30 | 19 |
| Fat | 13 | 10 | 21 | 16 |
| Animal fat | 8 | 6 | 11 | 9 |
| Vegetable fat | 4 | 3 | 8 | 6 |
| Saturated fat | 5 | 4 | 8 | 6 |
| Protein | 7 | 6 | 12 | 10 |
| Animal protein | 4 | 3 | 5 | 4 |
| Vegetable protein | 3 | 2 | 6 | 5 |
| Fibre | 1.3 | 1.1 | 3 | 2 |
| Alcohol | 0.7 | 0.3 | 1.1 | 0.5 |
| **Proportion of UPFs group** | | | | |
| (row percentage) | | | | |
| Year | 2005/2006 | | 2018/2020 | |
| Population Group | Adults | Elderly | Adults | Elderly |
| Consumed quantity | 6% | 4% | 8% | 5% |
| Energy (calories) | 14% | 11% | 26% | 20% |
| Carbohydrate | 16% | 13% | 34% | 26% |
| Sugar | 21% | 15% | 37% | 21% |
| Fat | 14% | 11% | 24% | 20% |
| Animal fat | 18% | 16% | 31% | 27% |
| Vegetable fat | 10% | 6% | 16% | 13% |
| Saturated fat | 18% | 15% | 31% | 26% |
| Protein | 8% | 7% | 14% | 12% |
| Animal protein | 7% | 5% | 10% | 8% |
| Vegetable protein | 12% | 10% | 24% | 22% |
| Fibre | 7% | 6% | 16% | 11% |
| Alcohol | 7% | 2% | 12% | 4% |

**
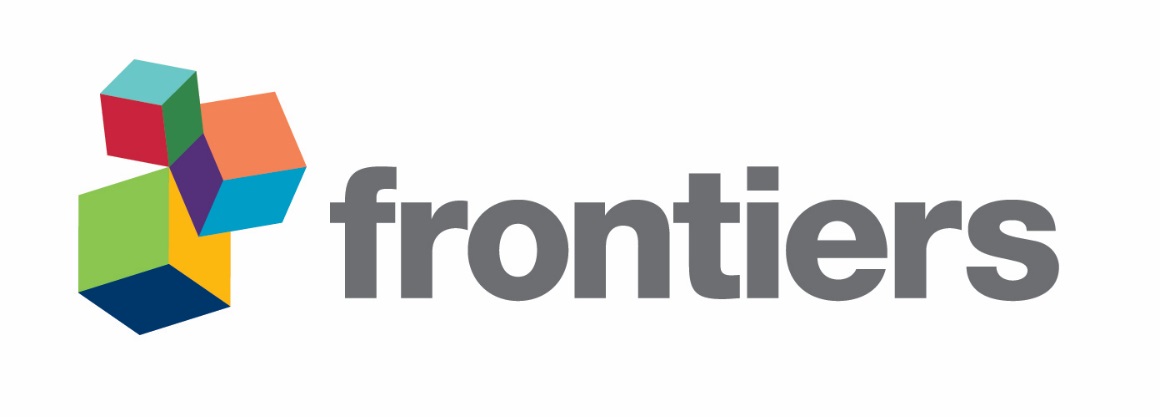
**
